# Geometrical design of 3D superconducting diodes

**DOI:** 10.1038/s43246-025-00788-1

**Published:** 2025-04-13

**Authors:** Philip JW Moll

**Affiliations:** Max-Planck-Institute for the Structure and Dynamics of Matter, Building 900, Luruper Chaussee 149, Hamburg, Germany

**Keywords:** Superconducting properties and materials, Surfaces, interfaces and thin films

## Abstract

The design of advanced functionality in superconducting electronics usually focuses on materials engineering, either in heterostructures or in compounds of unconventional quantum materials. Here we demonstrate a different strategy to bespoke function by controlling the 3D shape of superconductors on the micron-scale. As a demonstration, a large superconducting diode effect is engineered solely by 3D shape design of a conventional superconductor, ion-beam deposited tungsten. Its highly efficient diode behavior appears from its triangular cross-section when vortices break time-reversal and all mirror symmetries. Interestingly reciprocity is observed at four low-symmetry field angles where diode behavior would be expected. This can be understood as a geometric mechanism unique to triangular superconductors. Geometry and topology induce a rich internal structure due to the high-dimensional tuning parameter space of 3D microstructures, inaccessible to the conventional 2D design strategies in thin films.

## Abstract (web summary)

The design of advanced functionality in superconducting electronics is usually achieved by engineering bespoke heterostructures or identifying unconventional quantum materials. Here, an alternative strategy is demonstrated, whereby a large superconducting diode effect is engineered by the 3D micron-scale shape design of a conventional superconductor.

## Introduction

Currently, superconducting electronics are raising attention as quantum information processing on superconducting platforms is looming on the horizon. With quantum coherence as the new concept in circuit design, their unique characteristics as macroscopically coherent systems may finally overcome the economic challenges of their cryogenic temperature requirements. When, for example, qubits set (ultra-)low operation temperatures, exciting new opportunities even for classical superconducting circuit elements follow. In particular, the superconducting diode recently shifted into the research focus due to novel mechanisms of non-reciprocal charge transport compared to classical, single-electron electronics.

Superconductors support lossless dc-current flow up to its critical current, *I*_c_, above which resistance and dissipation set in. In a superconducting diode, the critical current magnitude depends on the current polarity, ${I}_{c}^{+}\ne {I}_{c}^{-}$. Such a device would offer ideal current rectification and direct signal propagation in superconducting electronics similar to diodes in ambient electronics. Fundamentally, superconductors that simultaneously break time-reversal and mirror symmetries may differentiate between forward and backward currents. Theory predicts these symmetry conditions in the bulk of exotic materials, for example, in finite momentum pairing systems^1–3^ or chiral SC that inherently break time-reversal symmetry^4^. Experimental observations have been reported in non-centrosymmetric crystals, such as the Dirac semi-metal NiTe_2_^5^, twisted trilayer graphene^6^, or heterostructures with varying spin-orbit coupling^7^.

While here, non-reciprocity heralds new insights into exotic superconductors, their diode efficiency is typically too low for practical applications. The efficiency is quantified as the normalized difference between the magnitude of the critical currents, $\eta =\frac{{I}_{c}^{+}-{I}_{c}^{-}}{{I}_{c}^{+}+{I}_{c}^{-}}$, ranging from zero (reciprocal transport) to one (ideal rectification). Here, superconducting vortex matter offers an established and optimized platform for the design of high-efficiency diodes under a weak external magnetic field. When the macroscopic shape of the superconductor breaks the mirror symmetry along the channel, and vortices break time-reversal symmetry, non-reciprocity is the norm, and diode behavior has been routinely observed for decades^8–10^. In this context, the term “asymmetric critical current” is used to denote SC diode behavior. Vortex entry and exit are highly dependent on the microscopic details of the surface barrier, such that minute and almost unavoidable fabrication imperfections suffice for diode behavior^11,12^. Thin-film devices with engineered asymmetry show sizable diode effects, for example, due to engineered boundary asymmetry, asymmetric anti-dot arrays^13^, superconductor/ferromagnet bilayers^11^, as well as Josephson junctions with individual trapped vortices^14^. The engineering challenge towards ideal vortex diodes thus is to create maximally left-right asymmetric channels and to tailor the non-linearity to match the requirements of real applications.

The physics of vortices and associated non-reciprocity is well established in thin-film (2D) and bulk (3D) geometries^15–20^. Here, we report an alternative regime of superconductors on a scale comparable to multiple penetration depths that are meaningfully three-dimensional. This denotes micron-sized wires with cross-section geometries that clearly differ from simple extrusions and, thus, truly are 3D objects. This concept is demonstrated in the specific case of a wire with a triangular cross-section, yet extensions to other geometric shapes naturally expand to further phenomena. Related triangular rods, hand-polished on the mm-scale, have already in 1965 been demonstrated by Swartz and Hart^8^ to be excellent SC diodes, albeit operating in a different physical regime of surface current flow owing to their macroscopic size and bulk pinning impeding vortex motion. Utilizing state-of-the-art microfabrication, controlling the cross-section shape becomes a powerful tuning parameter for vortex matter, and concepts from geometry naturally enter their physics as is here demonstrated in the triangular superconducting diode.

For a vortex system to be dominated by the 3D shape of the superconductor, the dominant force acting on vortices should be the energy barrier associated with their entry and exit, the Bean-Livingston barrier^21^. Akin to mirror charges in electrostatics, the surface provides a repulsive force hindering vortices from crossing it. Commonly, however, bulk defect pinning provides the dominant force in 3D objects^22^. Local defects suppress the condensation energy, which defines lower energy positions of vortices and thus impede their free motion and the associated dissipative voltage. Bulk pinning can either be minimized in ultra-clean crystals, or in ultra-disordered systems in which a high defect density compared to the vortex diameter effectively averages the pinning potential. We focus here on the recent observations of ultra-mobile vortices in Focused Ion beam (FIB)-deposited superconductors^23–25^. W-C-O is a highly carbon-rich amorphous superconducting deposit of tungsten^26^. It features a decent T_c_ ~ 5 K and upper critical field H_c2_(0 K) ~ 9.5 T, and accordingly one obtains the Ginzburg-Landau coherence length $\xi \left(0K\right) \sim 6\;{nm}$. The penetration depth is more difficult to infer in microstructures, yet using standard estimates from transport $\lambda \left(0K\right) \sim 650\;{nm}$ has been reported^27^. This high penetration depth arises from the high normal state resistivity and is in line with the limited mean-free path in this amorphous material. Tunneling spectroscopy^28^ unveils a fully gapped state with $\Delta \left(0K\right)=0.65 \, {meV}$, and a consistent picture of a conventional, strongly type-II superconductor deep in the dirty limit emerges.

This material can be conveniently deposited in a maskless direct-write technique on any substrate by FIB-assisted vapor deposition, which allows non-trivial shape control in 3D. Direct-writing of superconducting nanowires as well as functional devices such as superconducting quantum interference devices have been demonstrated^29–31^. The main observation reported here is the emergence of large superconducting diode effects with field-tunable polarity in such tungsten deposits. Exploiting the 3D fabrication capability of the FIB, inversion symmetry can be explicitly broken and tailored to asymmetric current-voltage characteristics designed as desired for applications requiring specific superconducting diodes.

## Results and discussion

### Broken inversion symmetry in wires of triangular cross-section

**Fig. 1:**
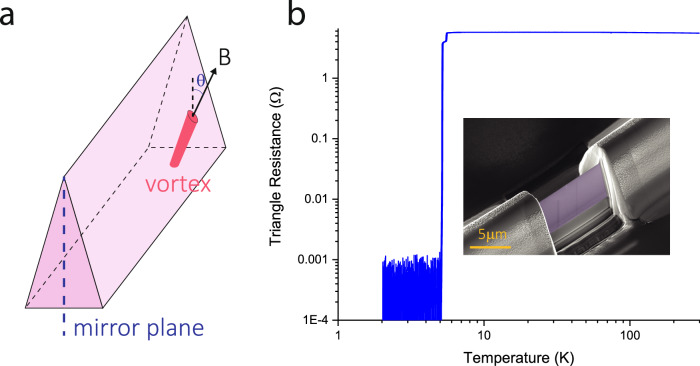
Triangular mesoscopic superconductor. **a** The active device is a straight wire with an isosceles triangular cross-section. Importantly, this shape features only one mirror symmetry that preserves the current polarity. The magnetic field angle, $\theta$, sets the direction of vortices and is measured from that mirror plane. **b** Inset: false-color SEM image of the real wire. The $10 \, \mu {m}$ long active section (purple) terminates in much bigger, bulky superconducting terminals to ensure the smallest critical current takes place in the triangular section. The isosceles triangle is $2.8 \, \mu {m}$ wide and $7.0\;\mu m$ tall, corresponding to an opening angle of 22°. Main: The resistance remains essentially independent down to *T*_c_ = 5.25 K. A weak double-transition appears due to a slightly higher *T*_c_ in the large SC contacts that were deposited at higher ion currents.

Here we focus on a $10 \, \mu m$ long wire with a cross-section precisely shaped as an oblate isosceles triangle ($2.8 \, \mu {m}$ wide, $7.0 \, \mu m$ tall, Fig. 1, see methods for fabrication details). The amorphous nature of the deposits is evidenced by the approximately temperature-independent resistance, placing them close to a metal-insulator transition. The SC transition, however, is sharp, and a noise-limited zero-resistance state is reached. To avoid complications of Lorentz force modulations or pinning effects at the contacts, we strictly keep the magnetic field perpendicular to the wire while rotating. The field-angle $\theta$ parametrizes the field direction, where $\theta ={0}^{\circ}$ corresponds to fields aligned with the tip of the triangle (Fig. 1).

**Fig. 2:**
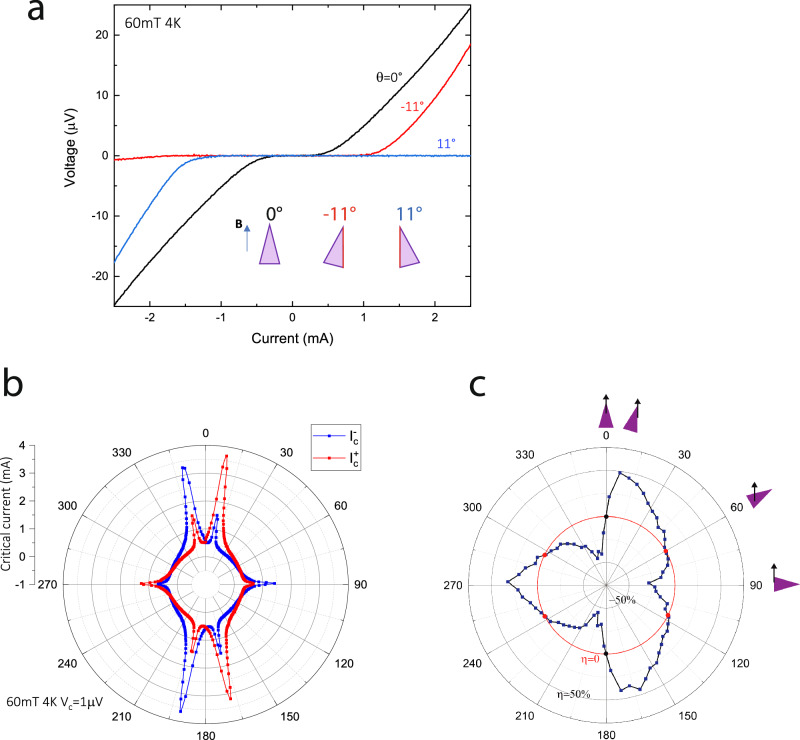
Superconducting vortex diode. **a** The current-voltage characteristics in the symmetric configuration (*θ* = 0°) is highly anti-symmetric while at shallow angles (±11°) pronounced diode effects are observed. In line with symmetry, the diode forward direction flips when rotating through $\theta ={0}^{\circ}$. **b** Over a full field rotation, the critical current is strongly modulated. The mirror symmetric appearance of ${I}_{c}^{+}$ and ${I}_{c}^{-}$ reflects how close the device is to the idealized triangle. Strong peaks occur at 6 angles with pronounced diode behavior. These correspond to special angles when the magnetic field is parallel to one of the triangle faces. **c** The diode efficiency $\eta$ computed at a voltage criterion ${V}_{c}=1 \, \mu V$ displays a distinct flower-shape associated with the triangle geometry. $\eta$ crosses zero (red circle) at 6 angles, two at the mirror-symmetric directions 0°,180°, and 4 times at low symmetry directions ${\theta }_{{top}}$ (and its symmetry copies).

When dominated by surface barrier pinning, such an object naturally acts as a highly efficient superconducting diode (Fig. 2), and the current polarity-conserving mirror plane of the geometric object plays a crucial role. When the magnetic field is applied within this mirror plane, that is $\theta ={0}^{\circ}$, symmetry dictates entirely anti-symmetric current-voltage characteristics (IV curve) as experimentally observed (black curve). Pulsed currents (1 ms duration) up to a range of 2.5 mA are used to avoid thermal effects in the data, as determined by self-heating tests (see methods). As the field is rotated out of this plane, the vortices explicitly and strongly break the mirror symmetry. Hence, within a narrow angle range, a massively asymmetric diode behavior emerges. This asymmetry finds a maximum at $\theta =11^{\circ }$, which corresponds to half of the geometric opening angle of the isosceles triangle studied here, $22^{\circ }$. Here the field is parallel to the left sidewall, which is a maximally asymmetric configuration of vortices entering either through a steep wall on one or through the knife-edge geometry on the other side. A large diode effect results as the current polarity sets the direction of the Lorentz force, ${F}_{L}\propto {j}_{s}\times B$, defining a vortex velocity perpendicular to the channel, and thereby which physical edge vortices enter. Moving *θ* = −11° aligns the field parallel with the opposite side, which inverts the diode from forward to backward. The polarity is such that positive currents define a leftward force looking along the wire (Fig. 2a), thus the vortex barrier is more effective in vortex exit than entrance processes. It is gratifying to see the nearly perfect asymmetry between these two directions, which evidences highly similar surface barriers on both faces as desired, which in turn demonstrates the achieved fabrication quality and reproducibility in this 3D structure. These results clearly demonstrate that highly asymmetric vortex barriers, and hence SC diodes, can be achieved with 3D shape control deterministically. Unlike thin films in 2D, the field direction plays a non-trivial role which does not only set the magnitude but also the forward direction of the diode. It follows naturally that the diode strength and field angle behavior can be designed as desired for a given application by changing the triangle shape. This sets such systems apart from engineered surface asymmetries in 2D, which are only sensitive to the out-of-plane component of the field when orbital effects are considered. Accordingly, in any 2D diode design, a field polarity change (or 180° rotation) is necessary to reverse the forward direction at a given current polarity. Control over arbitrary field angles for the diode strength and polarity reversal within a small angle range are new features uniquely enabled by the 3D nature of these devices.

### Continuous IV characteristics of 3D wires

Another important distinction of 3D bulk-like superconductors, as compared to thin films, is the sustainable flux flow without discrete avalanching or a thermal runaway-induced transition into the normal state. The here-reported IVs merge smoothly into a zero-voltage state and, thus, are not necessarily characterized by a sharp critical current. One may still define a critical voltage, either given by experimental resolution or concrete application demands, that implicitly defines a critical current as $V\left({I}_{c}\right)={V}_{c}$. For an initial glimpse at the data, one may trace such critical currents and the nominal $\eta$ as a rough measure of the diode strength over a full field rotation (Fig. 2b, c, ${V}_{c}=1 \, \mu V$). Beyond the rapid increase of $\eta$ up to 11°, a broad region of smoothly weakening diode strength follows. Eventually at 90°, $\eta$ changes sign as now the field is parallel to the short bottom face of the triangle. Due to the high symmetry of the problem, one quadrant (0°–90°) fully characterizes the device. Time-reversal symmetry ensures $V\left(I,B\right)=V\left(-I,-B\right)\equiv V(-I,\theta +{180}^{\circ }\, )$, hence $\eta \left(\theta \right)=-\eta (\theta +{180}^{\circ })$. Equally, the mirror symmetry enforces $\eta \left(\theta \right)=-\eta (-\theta )$, leading to symmetry-protected reciprocal transport ($\eta =0$) for fields in the mirror plane, $\theta ={0}^{\circ },{180}^{\circ }$, which is experimentally observed (Fig. 2c). This data demonstrates a high degree of structural tunability and design opportunities for desired field-angle behavior of SC diodes.

### Accidental reciprocity in low-symmetry configuration

Another interesting aspect concerns the absence of diode behavior at some specific oblique angles (red dots in Fig. 2c). Symmetry dictates reciprocity only for fields within the mirror plane, while at all other angles non-reciprocity is symmetry allowed and hence should be observed. This is true at all angles except at one additional zero crossing at ${\theta }_{{top}}\sim \,$72° (and its symmetry-related copies). The left-right asymmetry in a triangle generically inverts 6 times upon a full rotation, and hence it appears not surprising that a sign-changing real function, $\eta (\theta )$, can be fine-tuned to zero by carefully adjusting $\theta$ to some intermediate value.

Yet a single number $\eta$ is insufficient to describe the strongly non-linear diode behavior of such 3D structures. Its value depends on an arbitrary voltage criterion and thus has little merit in assessing neither the physical situation nor the technical relevance of the diode operating at certain bias levels. For example, carefully adjusting the experimental parameters and ${V}_{c}$ would, close to ${T}_{c}$, technically allow us to report record numbers of $\eta$ well above 90% with little practical relevance or insights. To better represent the non-reciprocity in these devices, we thus focus on the raw IV curves as customary in semiconducting diodes. Perfectly reciprocal conductance reflects in anti-symmetric IV curves, as $V\left(I\right)=-V(-I)$. Hence the symmetric voltage, ${V}_{{sym}}=\frac{1}{2}(V\left(I\right)+V\left(-I\right))$, provides a non-linear measure that directly encodes the diode strength at a current level $I$. Equivalently, the diode can be described in terms of a non-linear efficiency that explicitly depends on the voltage criterion used to define the critical current, $\eta ({V}_{c})$ (see methods).

**Fig. 3:**
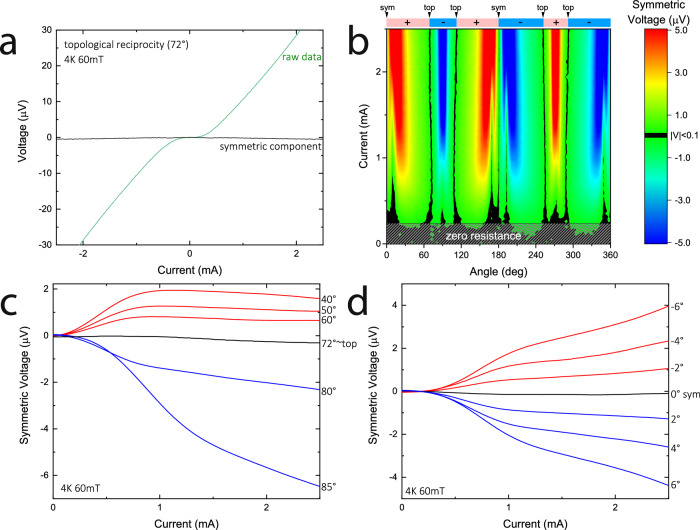
Symmetry and topology. **a** IV curve at the angle *θ*_top_ ~ 72° (@4 K, 60 mT) is highly anti-symmetric over the entire current range, despite the lack of symmetry at this angle. The symmetric component ${V}_{{sym}}$ is flat accordingly. **b** Scanning IV curves at the same conditions in 1° steps exposes the angular structure of ${V}_{{sym}}$. The diode direction changes six times over a full revolution, with reciprocal cases found equivalently six times (black lines, ${V}_{{sym}}=0$). **c**, **d** highlight the angular evolution of ${V}_{{sym}}$ (@4 K, 60 mT) crossing through the topological and symmetry-governed reciprocal angles. We associate the subtle deviations of ${V}_{{sym}}$ from zero at both angles with deviations of the real device from the idealized geometry.

The angle dependence of ${V}_{{sym}}$ further highlights the robustness of reciprocity at ${\theta }_{{top}}$ at all bias currents (Fig. 3a), as the IV curve is highly symmetric over the entire current range. The IV curves are higher-dimensional objects described by multiple fitting parameters, and hence it is remarkable that a complex curve ${V}_{{sym}}(I)$ can be tuned to nearly vanish by a single parameter. In the case of the symmetry-protected zero-crossings of $\eta$, this is enforced by conservation laws arising from time-reversal and mirror symmetry that hold at any bias (Fig. 3b). At the oblique angle ${\theta }_{{top}}$, no symmetry argument can be made, and reciprocity appears merely accidental (Fig. 3c). Note that ${V}_{{sym}}$ does not vanish exactly at ${\theta }_{{top}}$, with a similar residual magnitude compared to that in the symmetric orientation $\theta ={0}^{\circ }$. This small residual signal points to experimental deviations from the idealized case, such as non-ideal triangular shapes, thermal effects from weak but asymmetric self-heating, or field precession due to slight deviations of the triangular wire from the axis of rotation (Fig. 3d).

### Design principles of asymmetric vortex barriers

**Fig. 4:**
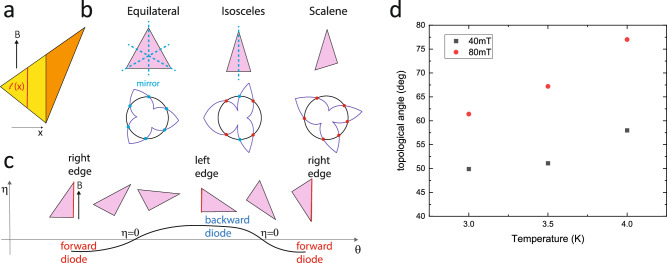
Evolution of geometry. **a** The triangular cross-section renders the length of the vortices following the field direction position dependent, $l(x)$, which forms the basis of the geometric force. At any angle except for the critical ones, the triangles decay into two regions (yellow and oriange) with ${F}_{{geom}}$ of different magnitude and opposite direction. **b** Each mirror symmetry constrains a pair of zero crossings. While the equilateral triangle pins all six crossings at mirror symmetry protected angles (blue dot), in the isosceles only two are set by symmetry, and in the scalene case all six crossings fall into the geometry enforced class (red dot). Exemplary plots of $\eta \left(\theta \right)$ are sketched, using the model presented in the methods. **c** The alternating left-right asymmetry is a natural consequence of any triangle, irrespective of its symmetries. **d** ${\theta }_{{top}}$ is strongly field and temperature-dependent, reflecting its decoupling from simple geometry.

To understand the geometric structure of the nonreciprocity from a vortex point-of-view, we first point to the importance of the special configurations when the magnetic field is parallel to one of the three faces of the triangle (Fig. 4). In the absence of bulk pinning, the well-controlled 3D shape results in a geometric force acting on the vortices. The flux lines are expected to follow the direction of the magnetic field given the microscopic wire geometry and, hence, the absence of notable demagnetization effects. The vortex length depends linearly on the position within the triangle. This causes a position-dependent potential energy of the vortex $U\left(x\right)=\alpha \epsilon {x}$, given a constant line energy $\epsilon$ per unit length and $\alpha =\frac{{dl}}{{dx}}$ denoting the geometric length change of the vortex. From this argument, a purely geometric force results, ${F}_{{geom}}=-\alpha \left(x\right)\epsilon$, which acts in addition to the Lorentz force ${F}_{L}$ on the vortices.

Any triangle decays into two regions of opposite sign of this geometric force (orange and yellow in Fig. 4a). These regions evolve as the triangle rotates in the magnetic field. As the vortex lines follow the magnetic field, $\alpha$ is tuned by the field angle. Importantly, $\alpha$ and thus the geometric force diverges at the special angles when the field is parallel to a triangle face, providing the highest barrier for vortex penetration. This picture is well supported by the angle dependence of ${I}_{c}\left(\theta \right)$, which shows pronounced spikes and sharp ${I}_{c}$ maxima at these geometric angles. In addition to an enhanced overall ${I}_{c},$ also a maximum in the diode effect is expected as observed reflecting the maximal left-right asymmetry of the geometric forces.

The sixfold alternation of the diode sign reveals an interesting geometric hierarchy of generic IV curves in triangular conductors, a unique result of 3D device design. In the most symmetric case of an equilateral triangle, each of its three mirror planes pins two crossings at multiples of 60°, $\theta ={i60}^{\circ} ,{i}=\{{\mathrm{0,1}},\ldots 5\}$ and symmetry exactly explains all reciprocal angles. Lowering the triangle symmetry removes two (isosceles) or three (scalene) mirror planes (Fig. 4b, c). Unpinned from the mirror planes, the crossing points at ${\theta }_{{top}}$ are free to move in angle but cannot disappear.

The overall flower-shaped $\eta \left(\theta \right)$ can be well captured by a simple geometric model based on the shape of the triangle alone, supporting the notion that the geometric surface barrier dominates over bulk pinning (see methods). While clearly microscopic modeling is required to explain the details of the nonreciprocity, the success of this outrageously oversimplified model further supports that geometry is the key factor in the device response. By design, the model predicts a zero crossing at the mirror-symmetry enforced angles, yet it clearly fails to predict the location of ${\theta }_{{top}}$ which further supports that it has no geometric meaning in the triangle. This is experimentally confirmed by its field- and temperature-dependence (Fig. 4d) which varies substantially from 77° at (80 mT,4 K) to 50° at (40 mT, 3 K), while the symmetry-enforced degeneracies remain constant within experimental resolution of the angle (±0.2°). Temperature tunes the vortex size and interaction, while the magnetic field sets their density. This, not surprisingly, impacts the non-linearity of conduction and, thereby, the IV curves. While these details move the location of the additional reciprocity, they cannot remove it.

### Classification of geometric and non-geometric reciprocity

To differentiate this additional reciprocal angle from geometri c reciprocity, we associate it with the topology of the triangle in contrast to geometrically defined reciprocity in mirror planes. Topology and geometry are intimately entangled in simple objects like triangles, and one may equally well discuss the observation of reciprocity at field angles not supported by symmetry entirely from a geometrical point of view. The force model in any triangular geometry predicts 6 sign changes of the diode efficiency, $\eta \left(\theta \right)$, in a full revolution which necessitates 6 zero crossings under the reasonable assumption of it being a continuous real function. Alternatively, one may consider the triangle as an element in the space of 2D polygons, spanned by continuous deformations of its vertices. This forms a proper equivalence class, and the topological invariant is the rather trivial Euler number in 2D, the number of edges (=3 for all triangles). This viewpoint introduces continuous paths of deformation between any arbitrary triangle to an equilateral one, in which all 6 reciprocal angles are pinned by mirror and rotation symmetries. Along this deformation path, sketched in Fig. 4b, these symmetries are smoothly broken, and the reciprocal angles detach from their high-symmetry positions. In this picture, the locations ${\theta }_{{top}}$ are smoothly connected to mirror symmetry-protected ones in the equilateral case, in which reciprocity is strictly enforced at all currents. Such a viewpoint of smooth deformations of the ${\theta }_{{top}}$ may be the basis of the surprising current range of reciprocity, with an entire curve ${V}_{{sym}}(I)$ approximately vanishing for both ${\theta }_{{sym}}$ and ${\theta }_{{top}}$ alike. As in any topological formulation of a condensed matter problem, the physics can be equally discussed in non-topological terms, here from an entirely geometric view.

## Conclusions

These results demonstrate that non-trivial 3D shapes open a rich door to engineering advanced electronic functionality and sought-after functionality even in traditional superconductors. Importantly, the 3D cross-section is a powerful yet thus far underutilized tuning space. The most studied platforms of thin (2D) or thick planar films (rectangular cross-sections) are of too high symmetry to exhibit non-trivial responses. For example, the inversion symmetry in ideal rectangular conductors, as the simplest 3D shape, does not support non-reciprocity at any field angle. With more complex 3D designs, diodes with bespoke field-angle response and desired current-voltage characteristics can be fabricated. In particular, the forward direction can be flipped by a small field-angle change, in stark contrast to the field polarity change required in 2D geometries, which may further lead to sensing applications. With 3D control providing a more complex, higher-dimensional geometric tuning space for these systems, non-trivial forms of geometry and topology naturally emerge. At the same time, additive fabrication methods such as FIB-deposition enable such 3D structures on a chip level, with a large design parameter space for potential applications. The magnitude of ${j}_{c},\,\eta$ can be designed and adapted to desirable operating field strengths (see methods). Changing the opening angle of the triangle, for example, allows adjusting the field-angle sensitivity of the device to desired higher (sensors) or lower (stable diodes) values. ${T}_{c}$ of W-C-O can be adjusted by ion currents and ion species, which adds another design dimension to optimized diodes. As currently the technological space of SC diodes is being explored, with ideas from low power rectification in quantum sensing to higher power rectification in leadless DC power transmission into quantum circuits, the design flexibility of 3D structures may prove a valuable asset in optimizing diodes towards specific applications.

It further provides a route towards rationally designing more efficient superconducting diodes, and probing putative upper bounds on supercurrent rectification. Clearly, 3D shape control proves to be a powerful engineering parameter for superconducting devices that deserves closer investigation.

## Methods

### Device fabrication

Tungsten deposition was carried out using a Ga-based focused ion-beam system (ThermoFisher Helios G4). A standard precursor gas, W(CO)_6_, was introduced into the chamber via the micro-nozzle of the ThermoFisher gas injection system. The substrate consisted of a sapphire chip with photolithographically patterned gold leads, which were grounded to mitigate charging effects during ion-beam exposure. Optimal deposition conditions were established at a beam energy of 12 kV and a dwell time of 50 ns. To minimize structural distortions caused by charging, a ~10 nm seed layer was initially deposited at a low current of 0.3 nA. Once the seed layer formed a robust connection to the electrodes, a stable discharge was observed, accompanied by a sharp, well-grounded image. For the main deposition, a high current of 20 nA was used to fabricate a rectangular structure measuring 100 μm in length, 5 μm in width, and 7 μm in height. Additionally, side contacts for four-probe voltage measurements were deposited, linking auxiliary gold pads to a 10 μm segment at the wire’s center, which would later form the triangular wire. These connecting structures were grown at 45 nA to expedite deposition. Due to the beam-condition dependence of superconducting parameters, these contacts exhibit a slightly different critical temperature (T_c_), manifesting as a weak double-step feature in the main Fig. 1.

The triangular wire was defined by FIB cutting at its center section. In the system used, the chip surface is oriented normally to the ion beam at a stage tilt of 52°. To achieve the desired 20° opening angle for the isosceles triangle, the stage was adjusted to 42°, and the cut was polished using a 30 kV ion beam at 300 pA. The targeted opening angle of 20° closely matches the experimentally determined 22° from magnetotransport measurements, with the slight deviation attributed to the finite beam width and the intrinsic opening angle of the cut.

### Geometric model

The full dynamics of the driven vortex system require a 3D simulation of the time-dependent Ginzburg-Landau model. This is complicated by the mesoscopic shape of the triangle (size $\sim 1-10\lambda$) and the non-negligible role of the geometric boundary. These challenging calculations will be an important step toward unlocking the engineering potential of 3D vortex matter. At the same time, it is gratifying to see how far arguments purely based on geometry, topology and symmetry go in explaining the complex data. These arguments can only yield, however, understanding at and closely around specific angles such as ${\theta }_{{top}}$.

**Fig. 5:**
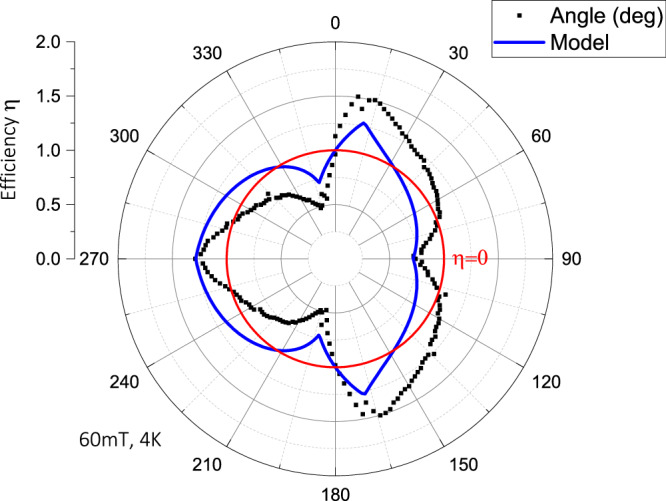
Comparison of the geometric model. to the measured ${{\rm{\eta }}}(1\,{{\rm{\mu }}}{{\rm{V}}})$ at 60 mT, 4 K.

Here, we develop a geometric model to assess the extent to which the anisotropy over a full angular rotation can be understood purely from geometry (Fig. 5). The model is phenomenological, and microscopic simulations are necessary to resolve finer details. Notably, it does not predict the location of the topological angle, reinforcing the idea that ${\theta }_{{top}}$ is not purely geometric but instead depends on temperature and field.

To capture the triangle geometry, one may be tempted to use the ratio of the geometric forces. Any triangle at arbitrary field angle decays into two regions in which the geometric force takes a different value with opposite direction. It is a straightforward geometric exercise to compute these two areas ${A}_{{right}}$ and ${A}_{{left}}$. The scaled difference between these areas $\widetilde{\eta }=c({A}_{{left}}-{A}_{{right}})/({A}_{{left}}+{A}_{{right}})$ is a natural proxy for the degree of imbalance between the forces. It obeys all symmetry requirements, for example, in the isosceles triangle $\widetilde{\eta }$ vanishes by design at the mirror symmetric configuration $\theta ={0}^{\circ }$ as both areas are equal.

This geometric construction (Fig. 5) reproduces the overall flower shape remarkably well, supporting that the surface boundary following the geometry is the dominant factor setting the non-reciprocity.

### Self-heating checks

**Fig. 6:**
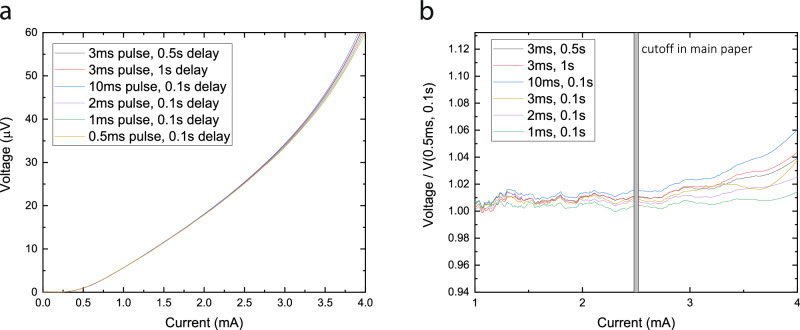
Self-heating tests. **a** Current pulse duration and delay variation to probe for self-heating effects. **b** Same data normalized by shortest applied pulse (0.5 ms). Below 2.5 mA, no effect of pulse duration and delay are observed. Above 2.5 mA, both delay and duration lead to signal deviation, signaling the onset of thermal effects and heat accumulation.

Thermal effects are a well-known source of hysteresis and non-linearity in superconducting elements, especially when high currents close to the critical current drive substantial power. To minimize thermal effects and eliminate thermal hysteresis, the triangles were probed using rectangular pulsed currents using 1 ms long pulses and an off-time of 100 ms, hence reducing the duty cycle to 1/100. The pulses were delivered using a Keithley 6221 Current Source, and voltages were recorded via a Keithley 2182 A Nanovoltmeter working in pulsed IV mode. Pre- and post-pulse voltages were averaged to compensate for the effects of drift and thermal voltages. Based on these tests, we limit the current range to 2.5 mA in the main paper and use conservative pulses of 1 ms at 0.1 s delay (Fig. 6).

### Diode efficiency ${{\boldsymbol{\eta}}}$

The commonly stated definition of diode efficiency, $\eta =({I}_{c}^{+}-{I}_{c}^{-})/({I}_{c}^{+}+{I}_{c}^{-})$, is a good description of the non-reciprocal conductance if the current driven transition into the normal state is very sharp. In that case, the region of zero resistance is sharply delineated from that of high, ohmic resistance. In many materials, including 3D systems, this transition is more gradual. Single vortex depinning leads to finite flux-flow resistance without a transition into an ohmic regime at or close to the normal state resistance. This sub-threshold behavior is highly non-linear, and operationally one picks a critical voltage, ${V}_{c}$, that demarks the onset of relevant dissipation levels for a particular application or physical phenomenon. The IV curves then implicitly define a critical current as the current needed to reach a voltage level set by this criterion, ${I}_{c}\equiv I({V}_{c})$. Again, if the IV curves are steplike or very sharp, the choice of criterion is not relevant. With broadened transitions reflecting the vortex behavior, this delineation is not sharp. Hence, in such non-linear cases, the diode efficiency strongly depends on the voltage criterion, $\eta ({V}_{c})$.

The current-dependence of the diode efficiency can be seen directly in the symmetric voltage, ${V}_{{symm}}=V\left(I\right)+V(-I)$, which vanishes in the absence of diode behavior and in magnitude gauges the asymmetry in voltage at a given current level. If one chooses, one can completely equivalently describe the situation from the viewpoint of a voltage criterion-dependent efficiency.

**Fig. 7:**
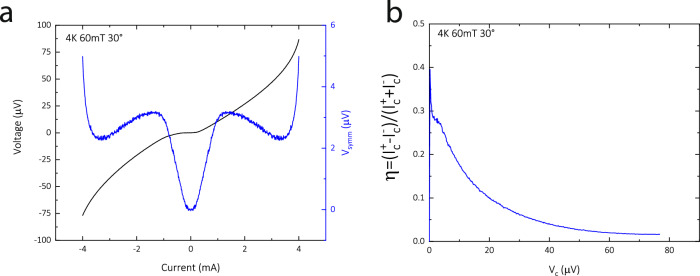
Voltage-dependent efficiency. **a** The raw IV data and symmetric voltage at an exemplary condition (4 K, 60 mT, 30°). Here, the diodes are most effective at low bias with a more symmetric behavior at high bias. **b** The corresponding voltage-dependence of the efficiency. As expected, this number is large at low dissipation and drops significantly as the system evolves to a high dissipation state.

In Fig. 7 we review an exemplary case of the translation into the language of criterion-dependent efficiency. Here, the cutoff voltage ${V}_{c}$ defines the critical currents and hence the efficiency at this one criterion. Choosing a lower one probes the higher asymmetry of the Bean-Livingston barrier for single vortex entry, while at high bias in a dynamic flow situation the importance of this barrier is reduced, leading to a more symmetric behavior.

### Temperature and magnetic field dependence

**Fig. 8:**
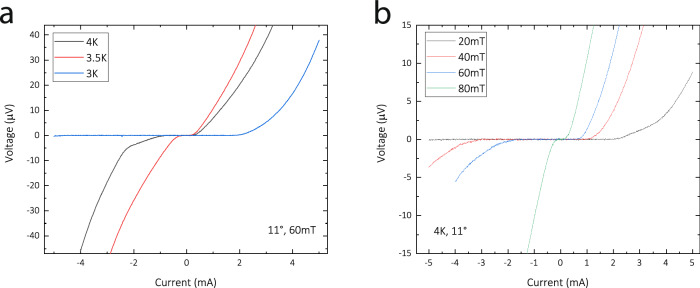
Field and temperature dependence. **a** Temperature dependence at fixed field of 60 mT for varying temperatures and **b** at fixed temperature of 4 K at varying fields. Note the extended current range of 5 mA, at which self-heating cannot be ignored. The currents were limited to 5 mA to limit thermal stress on the device and avoid subsequent pulse damage. At lower temperatures and fields, no more critical current was observed at negative (positive) polarity for a field angle of 11°(−11°).

The symmetry breaking effect of the magnetic field at oblique angles ensures diode behavior at any operating condition of the diode. The values of the critical currents, the shapes of the IV curves and accordingly ${V}_{{sym}}$ can be tuned by the field strength and the temperature. Figure 8 shows the IV curves at the optimal diode angle for this structure, 11°, at different magnetic fields and temperatures.

Diode effects at large efficiencies are observed under all conditions. In the main text, the discussion on symmetry focuses on a particular set of exemplary parameters, 60 mT and 4 K. This choice is out of practicality as the self-heating checks (see S3) have shown that at bias currents above 2.5 mA self-heating cannot be ignored. As in a SC diode the dissipation is asymmetric, self-heating will cause artificially enhanced diode behavior due to thermal runaway. This may be desirable in operando to obtain higher efficiencies; however, it complicates the discussion of the underlying physics. To discuss isothermal data, hence this parameter set was chosen. The operating space of the diode, however, is vast, and higher operating currents at lower temperatures show impressively large diode effects at high bias.

## Data Availability

All data are available on the repository zenodo at 10.5281/zenodo.15013112.
